# Numerical study of entrainment of the human circadian system and recovery by light treatment

**DOI:** 10.1186/s12976-018-0077-x

**Published:** 2018-05-09

**Authors:** Soon Ho Kim, Segun Goh, Kyungreem Han, Jong Won Kim, MooYoung Choi

**Affiliations:** 1 Department of Physics and Center for Theoretical Physics, Seoul National University, Gwanak-ro 1, Seoul, 08826 Korea; 2 Institut für Theoretische Physik II - Soft Matter, Heinrich-Heine- Universität Düsseldorf, Düsseldorf, D-40225 Germany; 3 Laboratory of Computational Biology, National Heart, Lung, and Blood Institute, National Institutes of Health, Bethesda, 20892 USA; 4 Department of Healthcare Information Technology, Inje University, Gimhae, 50834 Korea

**Keywords:** Mathematical model of sleep, Circadian rhythm, Entrainment, Orexin

## Abstract

**Background:**

While the effects of light as a zeitgeber are well known, the way the effects are modulated by features of the sleep-wake system still remains to be studied in detail.

**Methods:**

A mathematical model for disturbance and recovery of the human circadian system is presented. The model combines a circadian oscillator and a sleep-wake switch that includes the effects of orexin. By means of simulations, we characterize the period-locking zone of the model, where a stable 24-hour circadian rhythm exists, and the occurrence of circadian disruption due to both insufficient light and imbalance in orexin. We also investigate how daily bright light treatments of short duration can recover the normal circadian rhythm.

**Results:**

It is found that the system exhibits continuous phase advance/delay at lower/higher orexin levels. Bright light treatment simulations disclose two optimal time windows, corresponding to morning and evening light treatments. Among the two, the morning light treatment is found effective in a wider range of parameter values, with shorter recovery time.

**Conclusions:**

This approach offers a systematic way to determine the conditions under which circadian disruption occurs, and to evaluate the effects of light treatment. In particular, it could potentially offer a way to optimize light treatments for patients with circadian disruption, e.g., sleep and mood disorders, in clinical settings.

## Background

Circadian rhythms indicate a variety of biological oscillations which occur in living organisms with a period of approximately 24 h [1]. In mammals, the suprachiasmatic nucleus (SCN) located in the hypothalamus is the central pacemaker for circadian rhythms [2]. The SCN has a self-sustained, endogenous near-24 h period and gives cues to various functions of the body, acting as the master clock of the brain. In particular, the SCN is involved in the timing of the sleep-wake cycle, and applies increasing sleep pressure as the clock approaches subjective night [3]. The evolutionary advantage of the circadian pacemaker is its ability to allow the body to anticipate periodic events of the environment. The SCN thus adjusts its phase in response to the environment and is entrained to the daily cycle. The primary environmental cue that influences the phase of the SCN is the light-dark cycle. The retinohypothalamic tract transmits information on light intensity to the SCN [4, 5]. With daily adequate exposure to light, the entrained SCN ensures that we are active during the day and at rest during the night [6, 7]. The SCN along with the monoamine nucleus (MA), ventrolateral preoptic nucleus (VLPO), homeostatic regulators including adenosine, and orexenergic (ORX) neurons interact to consolidate a stable 24-h sleep-wake cycle [3, 8–11].

There are sleep disorders where circadian entrainment fails to occur [12]. When the entrainment fails, the SCN becomes desynchronized with the environment and the patient experiences drowsiness during the day and sleeplessness during the night. These sleep disorders often coincide with psychiatric disorders such as depression [13] or bipolar disorder [14], although the causal relation is not clear. To understand how the entrainment fails, one can conveniently consider a mathematical model and probe the mechanism via which circadian rhythm destabilization can occur in the model system. Indeed mathematical models for sleep-wake dynamics have long accompanied experimental discoveries [15–17]. The Phillips-Robinson (PR) model [18, 19] bases the sleep-wake system on a flip-flop switch between mutually inhibiting nuclei [10]. The PR model accurately describes core qualitative aspects of sleep, and can be fit to various experimental observations quantitatively. There have been studies examining responses of the model to external impulses [20] and noise [21]. It has also been extended in several ways to account for various phenomena related to sleep, including sleep deprivation [22], caffeine [23], narcolepsy [24], and shift work [25].

In this study we present an extension of the PR model, incorporating the effects of both orexin and light. We show that an imbalanced orexin level as well as insufficient light leads to a loss of the period-locked stable limit cycle in the system and that their effects are interdependent. Such combined effects have not been probed in previous studies, which considered only one or the other: Fulcher et al. examined how orexin relates to narcolepsy [24], but did not explore the effects of orexin on the circadian rhythm. Postnova et al. developed a circadian oscillator model and applied it to a shift work setting, but without effects of orexin [25]. Here, to obtain a more complete picture of circadian entrainment, we use the combined model and study the conditions under which entrainment occurs. Simulating light treatment, we also quantify the intensity threshold at which the entrainment is restored, as well as what time window leads to optimal treatment.

## Methods

### Mathematical model description

The model used in the current study is based on the PR model [18]. The core of the model is the flip-flop switch which arises from two mutually inhibiting neuronal populations, the VLPO and the MA. The average cell body potentials of these groups are represented by dynamical variables *V*_*v*_ and *V*_*m*_, respectively. In addition, we incorporate ORX neurons with cell potential *V*_*o*_, following the previous model study of narcolepsy [24]. The time evolution of the cell potentials is described by the coupled differential equations:

1 $$\begin{array}{*{20}l} \tau_{v} \frac{dV_{v}}{dt} &= -V_{v} + \nu_{vm} Q_{m} + \nu_{vh}H + \nu_{vc} C +A_{v}  \\ \tau_{m} \frac{dV_{m}}{dt} &= -V_{m} + \nu_{mv} Q_{v} + \nu_{mo} Q_{o}+A_{m}  \\ \tau_{o} \frac{dV_{o}}{dt} &= -V_{o} + \nu_{ov} Q_{v} + \nu_{oc} C + A_{o},  \end{array} $$

where *τ*_*j*_ is the characteristic time of population *j* (=*v*,*m*.*o*) and *ν*_*ij*_ (for *i*,*j*=*v*,*m*,*o*,*c*,*h*) measures the input from population *j* to *i*, with its sign indicating whether the connection is excitatory or inhibitory [26]. In general *ν*_*ij*_ is proportional to the average number of synapses from neurons of population *j* to neurons of population *i* [27]. Specifically, *ν*_*mo*_ is positive, reflecting the wake-promoting effect of orexin. The magnitude of *ν*_*mo*_, dubbed the orexin level, is interpreted to be the amount of orexin neurotransmitters in the brain.

The firing rate *Q*_*j*_ of population *j* takes the form

2 $$ Q_{j} =\frac{Q_{\text{max}}}{1+\exp\left[(\Theta - V_{j})/\sigma\right]},  $$

where *Q*_max_ is the maximum firing rate, *Θ* the mean firing threshold, and $(\pi /\sqrt {3})\sigma $ the standard deviation of the firing threshold. In Eq. (1), *A*_*v*_, *A*_*m*_, and *A*_*o*_ are constants while *H* and *C* denote the homeostatic sleep drive and the circadian sleep drive, respectively.

The homeostatic sleep drive *H* evolves according to

3 $$ \chi \frac{dH}{dt} = -H + \mu_{m} \frac{Q^{2}_{m}}{\eta_{h}+Q^{2}_{m}}   $$

with the characteristic time *χ*, which indicates that *H* increases during wake (*Q*_*m*_ large) and decreases during sleep (*Q*_*m*_ close to zero). The second term on the right-hand side, where *μ*_*m*_ and *η*_*h*_ are appropriate constants, describes the saturation behavior of *H* for larger values of *Q*_*m*_ [24].

The circadian rhythm has been modeled with a modified van der Pol oscillator that includes photic and non-photic influences [28]. This model has previously been combined with the PR model to model adaptation to shift work [25]. The equations for the circadian oscillator read

4 $$\begin{array}{*{20}l} \frac{1}{\Omega} \frac{dx}{dt} &= x_{c} + \gamma \left(\frac{1}{3}x + \frac{4}{3}x^{3} - \frac{256}{105} x^{7} \right) +B +N_{s}  \\ \frac{1}{\Omega} \frac{dx_{c}}{dt} &= {qBx}_{c} - x \left(\delta^{2}+kB \right),  \end{array} $$

which can be derived via the Liénard transformation of the Van der Pol equation [28]. Here *x* is the component of the circadian pacemaker that is directly related to the circadian sleep drive while *x*_*c*_ is a complementary variable. Their ratio gives the circadian phase *ϕ*_*x*_ according to tan*ϕ*_*x*_=*x*/*x*_*c*_. The characteristic frequency *Ω* scales the equation to the 24-h period and *γ* is the stiffness constant of the oscillator while constants *q* and *k* modulate the strength of the resetting effect of light via *B*. Finally, *δ* measures the intrinsic period of the oscillator relative to 24 h. Here the intrinsic circadian period of 24.2 h [29] leads to the value *δ*=(24×3600)/(24.2×3600×0.997)=0.994, where the correction factor 0.997 accounts for the nonlinearity of the oscillator.

The sleep drive *C* is defined simply to be *C*=*x* while the photic and non-photic influences *B* and *N*_*s*_ on the circadian oscillator are expressed as

5 $$\begin{array}{*{20}l} B &=G\alpha (1-n)(1-\epsilon x)(1-\epsilon x_{c})  \\ N_{s} &= \rho \left(\frac{1}{3}-s\right) \left[1-\tanh (rx)\right]  \end{array} $$

where *G*, *ε*, and *r* are constants and *n* is the fraction of photoreceptor cells that are activated. The first one of Eq. 5 has been chosen to characterize the varying sensitivity of the circadian oscillator to light throughout a day. Here *ρ* is the rate constant while *s* is the state variable taking the value unity (*s*=1) for the waking state and zero (*s*=0) for the sleeping state. It thus reads $s=\theta (V_{m} - V^{th}_{m})$, where *θ* is the Heavyside step function with the threshold mean MA potential $V^{th}_{m} = -2$ mV above which the system is defined to be in the waking state.

The rate of conversion from the ready state to the activated one of photoreceptors depends on the light intensity *I* via *α*:

6 $$  \frac{dn}{dt} = \alpha (1-n) - \beta n  $$

with

7 $$ \alpha = \alpha_{0} \left(\frac{I}{I_{0}} \right)^{p} \frac{I}{I + I_{1}},  $$

where *β* describes the rate of conversion from the activated state to the ready state. The form of *α* and constants *α*_0_, *I*_0_, *I*_1_, and *p* have been taken from [28] to fit the intensity response curve in high- and low-intensity ranges. Specifically, *α* increases with the light intensity in proportion to *I*^3/2^ at low intensities and to *I*^1/2^ at intensities much higher than *I*_1_. The light intensity *I*(*t*) affecting the photoreceptor cells is given by the environmental light $\widetilde {I}(t)$ along with the gating effect:

8 $$ I(t) = s \widetilde{I}(t).  $$

In this manner the photic driving force is gated by the sleep-wake state. Henceforth the tilde sign on $\widetilde {I}$ will be omitted for simplicity.

Note that Eqs. (4) to (8) are not present in Fulcher et al. [24], which adopted a fixed sinusoidal function to model the sleep drive *C*. This was adequate in that study, given their purpose of modeling narcolepsy, a state of unstable sleep-wake patterns. The addition of a circadian oscillator here allows us to probe the effects of orexin on a different kind of instability, circadian disruption. The circadian oscillator model was developed to respond realistically to broad ranges of the light intensity and duration. In particular, it describes accurately the human phase-response curve to light[28]. We have found that the phase-response curve remains nearly unchanged in the integrated model, with the sleep-wake circuitry added.

**Fig. 1:**
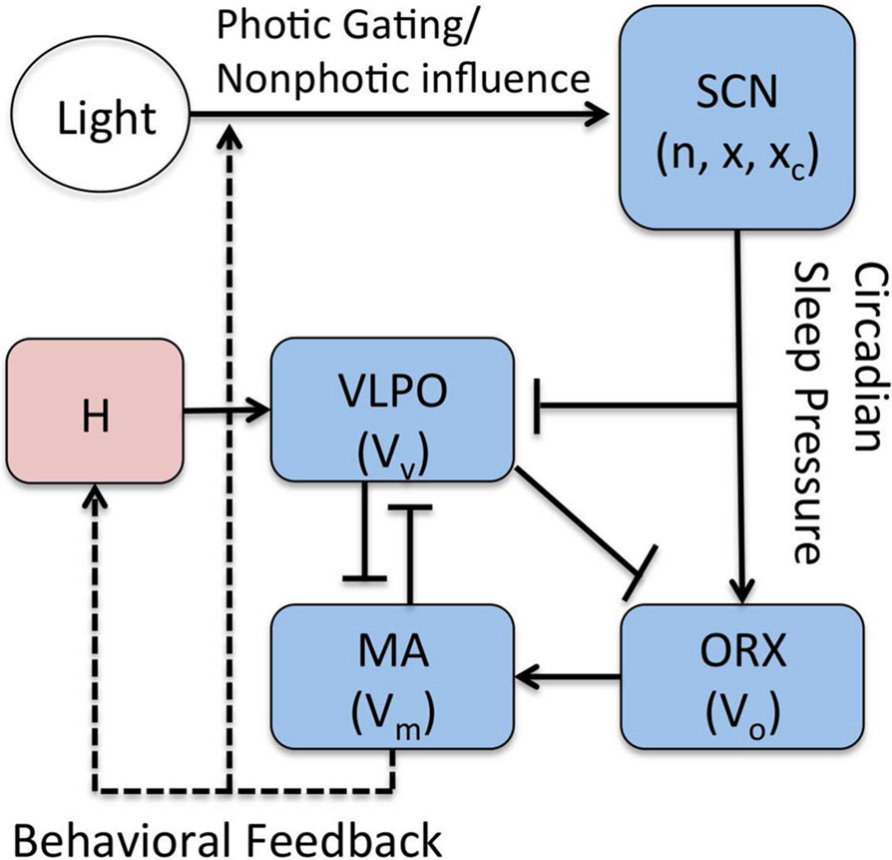
Schematic diagram of the sleep-wake model exhibiting components and their interconnections. Pointed-ends represent excitatory connections, while flat-ends represent inhibitory connections

The resulting sleep-wake model is illustrated schematically in Fig. 1, which exhibits the components of the model and interconnections between them. As given in the caption, pointed-ends represent excitatory connections between components whereas flat-ends represent inhibitory connections.

### Nominal parameters and light input

The nominal parameters used are shown in Table 1. Parameters involving the sleep-wake switch and homeostatic sleep drive have been taken from [24] and those involving the circadian oscillator taken from [25]. We further adjust a few parameters to produce realistic results, e.g., *χ*=40 h to produce a stable 8-h daily sleep bout with the 24-h period.

**Table 1:** Nominal parameter values of the model

Sleep-Wake Switch			Circadian Pacemaker		
Parameters	Values	Units	Parameters	Values	Units
*Q*_*max*_	100.00	s ^−1^	*Ω*	7.2722×10^−5^	s ^−1^
*Θ*	10.000	mV	*γ*	0.13000	−
*σ*	3.000	mV	*q*	0.60000	−
*ν*_*vm*_	− 2.1000	mVs	*k*	0.51000	−
*ν*_*mv*_	− 1.8000	mVs	*δ*	0.9944	−
*ν*_*vc*_	− 0.3000	mV	*β*	0.0070000	s ^−1^
*ν*_*vh*_	1.0000	mV	*α*_0_	0.100000	s ^−1^
*ν*_*oc*_	1.0000	mV	*p*	0.5	−
*ν*_*mo*_	0.30000	mVs	*I*_0_	9500	lux
*ν*_*ov*_	− 1.0000	mVs	*I*_1_	100	lux
*τ*_*v*_,*τ*_*m*_	10	s	*G*	2220.0	s
*τ*_*o*_	120	s	*ρ*	0.032000	−
*χ*	40.000	h	*ε*	0.40000	−
*μ*_*m*_	17.000	−	*r*	10.000	−
*η*_*h*_	2.3000	s ^−2^	*I*_*d*_	600	lux
*A*_*v*_	− 8.5000	mV	*I*_*n*_	150	lux
*A*_*m*_	0.52000	mV	*T*_*d*_	11.000	h
*A*_*o*_	1.0000	mV			

The environmental light is modeled as a simple square waveform:

9 $$ I(t) = \left\{\begin{array}{ll} I_{d}\ &\text{for}~~ 12\,\mathrm{h} -T_{d}/2 \leq t^{*} < 12\,\mathrm{h} +T_{d}/2 \\ I_{n}\ &\text{otherwise},  \end{array}\right.  $$

where *t*^∗^ is defined to be *t* modulo 24 h and corresponds to the day time. Accordingly, *I*(*t*) is given by a 24 h-periodic square function with *T*_*d*_ hours of daylight of intensity *I*_*d*_ and 24−*T*_*d*_ hours of dim light of intensity *I*_*n*_. The dynamical equations are then periodic with period 24 h, corresponding to the 24-h day-night cycle. For our nominal parameter set, we use *I*_*d*_=600 lux, *I*_*n*_=150 lux, and *T*_*d*_=11 h (see Table 1).

**Fig. 2:**
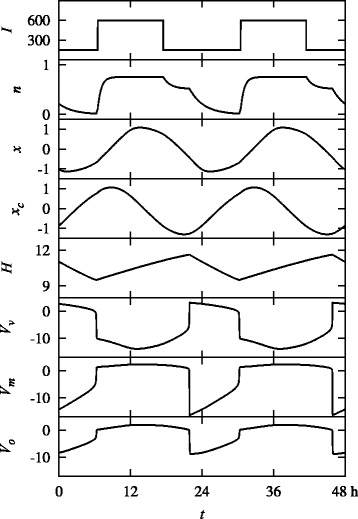
Plot of model dynamics during 48 h for the nominal parameters in Table 1. The light input *I* (in units of lux), having a 24-h period, results in a stable 24-h sleep-wake cycle, as manifested by the behaviors of seven dynamical variables: *n*, *x*, *x*_*c*_, *H* as well as cell potentials *V*_*v*_, *V*_*m*_, and *V*_*o*_ (in units of mV)

Figure 2 exhibits the resulting dynamics of the model. Specifically, the time evolutions of seven dynamical variables: the fraction *n* of activated photoreceptor cells, circadian oscillator variables *x* and *x*_*c*_, sleep drive *H*, and cell potentials *V*_*v*_, *V*_*m*_, and *V*_*o*_ of VLPO, MA, and ORX neurons, respectively, are plotted, together with the input light intensity *I*, during 48 h.

For a later purpose, we also remark that the circadian phase is related to the core body temperature (CBT) minimum via [28]

10 $$ t_{\text{CBT},i} = t_{\phi,i}+ t_{d},  $$

where *t*_CBT,*i*_ is the time of the CBT minimum on the *i*th day and *t*_*ϕ*,*i*_ is the time at which the circadian phase takes the value *ϕ*_*x*_=−170.7° on the same day *i*. The time lag between the two is given by *t*_*d*_=0.97 h. The average phase shift between CBT minima of successive days is then defined to be

11 $$  \Delta \equiv \frac{1}{N-1}\sum_{i=1}^{N-1} \left[t_{\text{CBT},i{+}1} -t_{\text{CBT},i}\right],  $$

where *N* is the total number of days of concern.

## Results

### Period locking zone

We now study the dynamics of the model by means of simulations, employing the 4th-order Runge-Kutta method. Numerical integration of the equations describing the model with the nominal parameters in Table 1 results in a stable 24-h limit cycle, which is interpreted as successful light entrainment: The circadian oscillator is coupled well to the light Zeitgeber, via the term *B* in Eq. (4), and the circadian oscillator in turn fixes the sleep-wake cycle [Eq. (1)], so that sleep occurs during the dark phase and wake occurs during the light phase.

Performing simulations in the absence of light (*I*_*d*_=*I*_*n*_=0), we find that the system exhibits a limit cycle with a period of 24 h and 22 min. Hence if the strength of light is weakened, the intrinsic period of the system will overcome the effects of light and there will be a constant phase delay. The critical daylight intensity at which the stable state disappears corresponds to the period-locking bifurcation point.

On the other hand, when the orexin level *ν*_*mo*_ is varied, this intrinsic period is altered by the change in the phase and length of the non-photic influence *N*_*s*_: A higher values of *ν*_*mo*_ leads to a longer period. This is an alternate way in which stability can disappear.

**Fig. 3:**
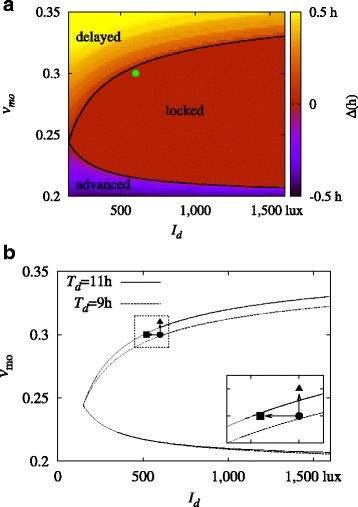
**a** Average shift *Δ* of the CBT minimum, obtained via simulations with *T*_*d*_=11 h, in the *ν*_*mo*_- *I*_*d*_ parameter space. **b** Period-locking zone boundaries for two different values of *T*_*d*_. Points are marked for normal (circle), seasonal disorder (square), and non-seasonal disorder (triangle). The arrows indicate shifts from the normal state to the disordered states (see the text). Note that in simulations the leftward arrow (shift to seasonal disorder) is accompanied by a change in *T*_*d*_ from 11 h to 9 h. The area enclosed in the the dotted box is enlarged in the inset

We carry out simulations of the model using a range of values of *ν*_*mo*_ and *I*_*d*_. To be specific, we sweep the *ν*_*mo*_- *I*_*d*_ parameter space, and take values of *ν*_*mo*_ ranging from 0.2 mV s to 0.35 mV s with increments of 0.0002 mV s and of *I*_*d*_ from 150 to 16,000 lux with increments of 10 lux. For each pair of parameters, simulations have been performed for the duration of 60 weeks, of which the data for the first 20 weeks are discarded for equilibration. Accordingly, the average phase shift *Δ* in Eq. (11) is obtained via averaging over the last 40 weeks (i.e., *N*=280 days). The resulting heatmap plot of the phase shift *Δ* is given in Fig. 3a. The red region indicates the period-locked zone, where no phase shift arises (*Δ*=0).

The boundaries of this zone are depicted in Fig. 3b for two different values of daytime duration *T*_*d*_. When the total exposure to bright light is low as in winter (*T*_*d*_=9 h), the stability region is observed to be reduced appreciably. On the other hand, extended duration of daytime (*T*_*d*_=11 h) tends to widen the stable region.

Figure 3 manifests that there are three routes to the loss of the 24-h period: (1) by decreasing the daytime duration *T*_*d*_, which shrinks the area of the period-locked zone; (2) by lowering the daylight intensity *I*_*d*_, which amounts to moving left on the *ν*_*mo*_- *I*_*d*_ parameter plane; and (3) by changing *ν*_*mo*_, which corresponds to moving up or down on the parameter plane.

The sleep-wake system maintains a stable 24-h cycle by means of its phase-resetting response to light. When daylight is insufficient, the photic driving force is not enough for entrainment to occur. This is the case in routes (1) and (2) above. However, route (3) shows that an imbalance in orexinergic neurons can cause circadian disruption even when daylight is typically sufficient. The mechanism by which this occurs is due to the wake-promoting nature of ORX. When *ν*_*mo*_ is increased, sleep and wake onset times are delayed with respect to the phase of the circadian oscillator. Due to the gating effects present in the photic drive *B*, this causes more light to enter the system at subjective night and less to enter in the subjective morning. The human phase response curve is such that morning light causes phase advance and evening light causes delay. In consequence the increase in ORX causes phase delay. Similar effects are observed when a constant excitatory stimulation term is added to ORX; this may be achieved simply by increasing the constant *A*_*o*_.

### Diseased states and light treatment

The above results show that both lack of daylight and high orexin levels can cause destabilization in the timing of the onset of sleep. The former is thought to be the case in seasonal affective disorder while the latter is regarded as a possible mechanism for non-seasonal affective disorder. Here we examine the effects of light treatment on such diseased states.

We first simulate bright light treatment in the case of insufficient light, which consists in artificial exposure to strong white light for a short time (e.g., 1.5 h). Simulations begin in the limit cycle of the model with nominal parameters; then the daylight length *T*_*d*_ and intensity *I*_*d*_ are reduced linearly, from 11 to 9 h and from 600 to 520 lux, respectively. This is intended to simulate a rapid change into winter light conditions, inducing circadian phase shifts to become increasingly misaligned with the light input.

We then consider the treatment by applying light of intensity *I*_tr_ in addition to the underlying (environmental) light in Eq. (9). During four days into full winter, light treatment is applied daily by taking *I*_tr_=10,000 lux for an hour and half each day. Light treatment protocols vary across studies; here the intensity and duration of light has been adopted from the study of light treatment as an antidepressant [30]. Treatment begins at time *t*_0_ of each day, which we set to 7 am unless stated otherwise. If the time *t*_CBT_ of the CBT minimum drifts towards a stable time within an hour from its initial value, we consider the system to be recovered.

In winter simulations, the wake onset time is retarded under the winter conditions and becomes desynchronized with light in the absence of light treatment. When light treatment is applied, the wake onset is advanced from the delayed phase and settles into the value near its initial phase of 10 pm, indicating recovery.

Next, we simulate a diseased state under normal light circumstances, where the instability is caused by an abnormal value of *ν*_*mo*_. We see in Fig. 3 that there are two ways in which this can happen: one by a large value of *ν*_*mo*_, where sleep timing is constantly delayed, and the other by a low value of *ν*_*mo*_, where sleep is advanced. Here we illustrate the former case only.

**Fig. 4:**
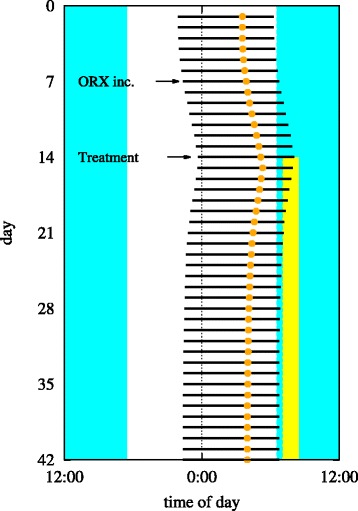
Daily sleep times (black lines) and CBT minima (colored dots) during simulations of light treatment on non-seasonal affective disorder. The orexin level is increased on day 7 (indicated by an arrow), causing instability. Starting on day 14 (indicated by another arrow), morning light treatment of 10,000 lux for 1.5 h daily is administered and maintained during the times labeled yellow, which causes a gradual return to the normal phase

Figure 4 presents the result of the non-seasonal case. Starting on day 10, we raise *ν*_*mo*_ from 0.3 to 0.31 mV s, which lies above the stable zone in Fig. 3a, while keeping *I*_*d*_ and *T*_*d*_ in their nominal values. As in the seasonal case, stability is restored when light treatment is applied and the system returns to a normal sleep-wake cycle. Thus the model shows that light treatment has stabilizing effects even when the instability does not arise from the lack of environmental light.

### Optimal light treatment times

**Fig. 5:**
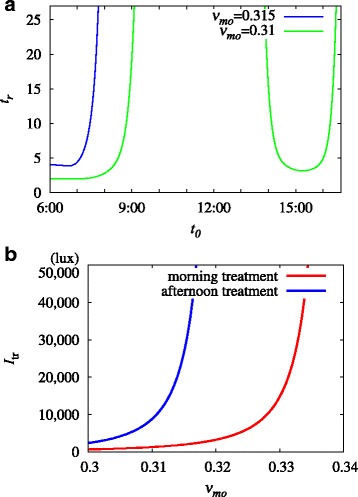
**a** Recovery time *t*_r_ (in units of day), i.e., days of treatment required for recovery from the circadian instability caused by orexinergic imbalance, versus treatment timing *t*_0_, for two values of *ν*_*mo*_. Interpolation has been used to produce a smooth curve. **b** Phase diagram on the *I*_tr_- *ν*_*mo*_ plane, for morning treatment (*t*_0_=7 am) and afternoon treatment (*t*_0_=3 pm). Above the curves, recovery occurs

We now explore how the efficacy of bright light treatment depends on the timing of treatment. Figure 5a demonstrates the sensitive dependence of the recovery time *t*_r_, i.e., the time duration of treatment required for a return to the initial phase, on the beginning time *t*_0_ of light treatment in the case of non-seasonal depression, for two values of *ν*_*mo*_. Note that in the case of *ν*_*mo*_=0.31 mV s, recovery occurs in the two limits of the treatment timing: one in the morning and the other in the afternoon. When *ν*_*mo*_=0.315 mV s, the recovery time for morning recovery is increased while afternoon recovery ceases to work.

It is suggested in Fig. 5a that in the case of phase-delay instability, both morning and evening bright light treatments are effective although morning treatment will be efficacious in a larger range of parameters. To make clear the difference in the efficacy between morning and afternoon treatments, we select two representative values of *t*_0_ corresponding to morning (*t*_0_=7:00) and afternoon (*t*_0_=15:00) treatments. For each treatment time, we calculate the light treatment intensity *I*_tr_ at which recovery occurs for varying orexin levels.

This leads to a phase diagram on the *I*_tr_- *ν*_*mo*_ plane, which is shown in Fig. 5b. It is observed that for all values of *ν*_*mo*_, the required treatment intensity is much larger for afternoon treatment. It is also observed that the required treatment intensity *I*_tr_ grows rapidly with *ν*_*mo*_. Specifically, at *ν*_*mo*_=0.32 mV s, the intensity *I*_tr_ becomes unrealistically large, indicating that only morning treatment is feasible. As *ν*_*mo*_ is increased further, e.g., to *ν*_*mo*_=0.34 mV s, this light treatment scheme becomes unfeasible at any intensity.

### Effects of noise

Noise is an important aspect of a realistic biological system. Here we consider the effects of noise by modifying the VLPO and MA equations of Eq. (1) in the following way:

12 $$\begin{array}{*{20}l} \tau_{v} \frac{dV_{v}}{dt} & = -V_{v} + \nu_{vm} Q_{m} + \nu_{vh}H + \nu_{vc} C +A_{v} + D \xi_{v}(t), \\ \tau_{m} \frac{dV_{m}}{dt}& = -V_{m} + \nu_{mv} Q_{v} + \nu_{mo} Q_{o}+A_{m} + D \xi_{m}(t),  \end{array} $$

where the added terms *ξ*_*j*_ (*j*=*v*,*m*) are Gaussian white noise characterized by 〈*ξ*_*j*_(*t*)〉=0 and 〈*ξ*_*j*_(*t*)*ξ*_*j*_(*t*^′^)〉=*δ*(*t*−*t*^′^) with noise strength *D*. Following existing studies [24, 31], we choose not to add noise to the ORX equation; we expect that doing so would bring additional noise in the MA neurons and not affect significantly the results.

Starting from the periodic solution, we perform simulations for 90 days, so as for the circadian system to settle possibly into its new equilibrium. We then simulate additional 40 days and observe whether circadian phase shifts occur. This process is repeated 50 times with new random seeds. Initially, the noise level is taken to be *D*=0.01 mV, and the entire process is repeated with *D* increased in increments of 0.01 mV.

**Fig. 6:**
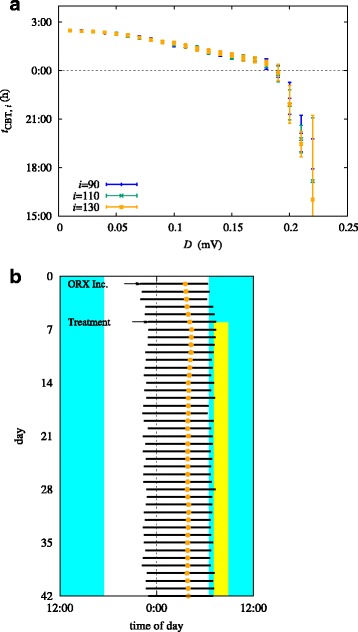
**a** Core body temperature minimum time *t*_CBT,*i*_ versus noise level *D*, obtained from simulations. The averages and standard deviations are plotted for the 90th, 110th, and 130th days (blue, green, orange) for *D*=0.01 mV to 0.25 mV. **b** Light treatment simulations for *ν*_*mo*_=0.33 mV s, treatment time *t*_0_=7:00, and *I*_*tr*_=10,000 lux, in the presence of noise *D*=0.1mV. As in the case without noise, recovery to the normal phase is observed

Figure 6a shows the distribution of the CBT minimum time *t*_CBT,i_ over the last 40 days of simulations for the range of *D* considered. It is observed that the circadian phase shifts to earlier timings as the noise level is increased. Moreover, noise tends to provoke the CBT timing (specified by *t*_CBT,i_) to spread: For instance, the standard deviation of *t*_CBT,i_ takes the value of about 7 min at *D*=0.1 mV. When the noise level is low (*D*<0.21 mV), the system settles into a new equilibrium within 90 days and the distribution of *t*_CBT,i_ does not change significantly over the next 40 days. In other words, periodicity, albeit fluctuating, is preserved. At *D*=0.21 mV, however, there appears a slight advance, which, for *D*>0.21 mV, increases substantially; this indicates that circadian disruption occurs at *D*=0.21±0.01 mV. When *D* is increased further to the order of 1 mV, noise dominates the sleep-wake switch and drives the system to switch erratically between sleep and wake.

Due to the phase advance effect described above, we expect the stability landscape of Fig. 3a to change with the introduction of noise. Setting *D*=0.1 mV, we perform simulations with *ν*_*mo*_ varied about its nominal value in increments of 0.001 mV s, and find that circadian entrainment occurs in the range 0.244 mV s≤*ν*_*mo*_≤0.321 mV s, below/above which continuous phase advance/delay is observed. This range is to be compared with that in the absence of noise (*D*=0 mV), namely, 0.229 mV s≤*ν*_*mo*_≤0.305 mV s. Such a shift of the stability region toward higher orexin levels indicates that the addition of noise offsets some of the phase-delaying effects of orexin. Finally, we simulate morning bright light treatment with *D*=0.1 mV, *ν*_*mo*_=0.33 mV s, *t*_0_=7:00, and *I*_*tr*_=10,000 lux, to find that the system undergoes recovery to the normal phase [see Fig. 6b].

## Discussion

The results of the integrated sleep-wake model provides a mathematical basis for many results established as to circadian entrainment. For example, many blind individuals experience a cyclic sleep disorder: They experience normal sleep-wake behavior for days to weeks at one time, followed by difficulty in sleeping at night and staying awake during the day for a period of time. Observation of such patients discloses that they experience continuous phase delay [32, 33]. Such patients are not entrained to light because they lack stimulation via the retinohypothalamic tract. Assuming nominal parameters otherwise, we should expect behavior corresponding to the upper left-most region in Fig. 3a, which does indicate continuous phase delay.

In sighted individuals, failure of entrainment results in non-24-h sleep-wake syndrome, similar to the above case. On the other hand, there are cases where the circadian phase is period-locked but at an abnormal time. This is the case in delayed sleep phase syndrome or advanced sleep phase syndrome, where the patient has a 24-h circadian rhythm but with a phase significantly late or early relative to the socially acceptable time. Namely, the system locates on the right of the curves in Fig. 3, but has a late or early phase due to an abnormal orexin level or inadequate exposure to light. Previous studies reported that delayed sleep phase syndrome arises in both circadian and sleep homeostatic systems [34]; our model offers a way to examine the interplay of those contributions, and although this issue is not explicitly explored in this study, it should be explored in the future.

Circadian rhythm disruption is known to be important in seasonal affective disorder, where change in the intensity and duration of light exposure is involved [13, 35]. In our model, we have seen that this corresponds to moving to the left on the parameter plane of Fig. 3b and shrinking the period-locking curve. The restoration of entrainment via bright light treatment as seen in our model is a possible mechanism behind the reported efficacy of bright light treatment as an antidepressant in seasonal affective disorder [35, 36]. Note here that lack of light exposure is not the only cause of circadian instability and that there is there is also evidence for the effectiveness of the bright light therapy in treatment of nonseasonal mood disorders [37, 38]. Relatedly, ORX neurons are implicated both in sleep disorders and in mood disorders [39]; our model study has shown that circadian disruption is a channel through which these effects may occur.

Our results thus demonstrate two different channels through which circadian disruption can occur: lack of light and orexin imbalance. Moreover, it shows that bright light treatment can be effective in restoring a normal circadian rhythm in both cases. In the case of orexin imbalance, Fig. 5 shows that the there are two time windows during which bright light treatment is effective, with morning treatment being effective for a wider range of circumstances. This result is consistent with the fact that morning bright light treatment is generally more effective than evening treatment in clinical studies. For example, Avery et al. [40] studied changes in the Hamilton Rating Scale for Depression (HRSD) scores after bright light treatment in winter depression, and found that morning treatment resulted in significantly higher remission rates compared with evening treatment. With refinement, our approach can be used to predict the efficacy of bright light treatment in specific circumstances and to guide practical applications. In that regard, it would be desirable to have more rigorous fitting to experimental results, based on, e.g., systematic investigation of the noise effects on entrainment conditions and the efficacy of bright light treatment.

## Conclusion

A model study was presented investigating circadian entrainment, disruption, and recovery. This study is the first attempt at a combined framework that models the effects of light and orexin on the entrainments. The approach provides a way to determine the conditions under which circadian disruption occurs, to evaluate the effects of light treatment, and to identify optimal treatment times. Light treatment methods could be costly, and it would be desirable to determine specifically the optimal time of day and the required intensity of light in applying the therapy. The results are consistent with light treatment data, and make a first step towards useful methodology for light treatment determination. To determine optimal light treatment for actual patients using this model, one needs to calibrate various parameters more precisely and to take individual variations into consideration; this is left for future study.
